# Entrepreneurship for People With Disabilities: From Skills to Social Value

**DOI:** 10.3389/fpsyg.2021.699833

**Published:** 2021-07-07

**Authors:** Pilar Ortiz García, Ángel José Olaz Capitán

**Affiliations:** Sociology Department, University of Murcia, Murcia, Spain

**Keywords:** disability, competencies, entrepreneurship, social value, autonomy

## Abstract

Entrepreneurship has undoubted social value as it contributes to socio-economic development of the context where entrepreneurship takes place. When the entrepreneurial activity is undertaken among especially vulnerable groups in the labor market, the multiplying effect of this value is made explicit in society, in general, and in the collective of people with disabilities (PWDs), in particular. The objective of this research study is to explore under which conditions this happens through the analysis not only of the relationship between the competencies that PWDs attribute to themselves and their development of the entrepreneurial activity but also of that between entrepreneurship and certain conditions that potentially create value by increasing the autonomy among this collective. A quantitative methodology based on the analysis of the survey carried out on a sample of 224 entrepreneurs with physical, sensory, or organic disabilities throughout Spain has been used. According to the results, entrepreneurs with disabilities (EWDs) have a higher self-evaluation competency. Furthermore, significant results concerning the link between the form of autonomous cohabitation of this collective and entrepreneurship have been obtained.

## Introduction

Since 1997 (the year of the Luxembourg Summit where agreements on the reduction in unemployment at the European level were adopted), the European Employment Strategy (EES) has played an important role in guiding the employment policies of the member states. Currently, this initiative is part of the Europe 2020 growth strategy. Within the implementation of this program, in the case of Spain, various initiatives, which are based on strategies that combine the flexibility required by the current production system with the security of a quality job, have been undertaken to combat unemployment and/or promote employment. This is especially important for vulnerable groups in the labor market, such as people with disabilities (hereinafter, PWDs). However, such policies have not been exempt from some criticism. Authors such as Crespo and Serrano (2013) have analyzed the latent function of European policies, whose aim is to construct a “common orientation” about employment based on the notions of employability, entrepreneurship, activation, and other synonyms that place the responsibility for success in the labor market on the individual. This strategy leaves in a situation of vulnerability collectives such as PWDs, who have special difficulties in finding employment, either as employees or as employers due to their peculiarities, if they are constructed by society (Berger and Luckmann, 1966) or determined by their physical or mental conditions.

The coronavirus disease 2019 (COVID-19) crisis occurred after suffering the last economic crisis (2008–2014) deteriorating even more the already precarious employment situation of this collective (Manzanera and Ortiz, 2017). In this context, the entrepreneurship of PWDs may represent an opportunity for labor and social inclusion in line with inclusive public policies that promote their autonomy (Jones and Latreille, 2011; Toboso and Rogero, 2012; Moreno, 2019). Fostering the autonomy that entrepreneurship requires, in the case of PWDs, not only mitigates the disadvantages they face, namely their type of disability and the degree to which it manifests itself but also taking into account the other sociodemographic factors that limit the aforementioned autonomy, such as age, level of education, or geographic location (Lindsay et al., 2015).

Until now, the study of entrepreneurship in PWDs in terms of competencies is unusual in the field of entities and organizations of the social sector, both in collective and associative manifestations of entrepreneurship, so it is necessary to resort to the competencies that they are self-attributed and that can encourage the group of PWDs.

It is statistically evident that entrepreneurship is lower among PWDs than among people without disabilities. According to the latest data provided by the Statistics National Institute of Spain (INE, 2020: 1–3), in 2019 there were 20,191,876,900 PWDs of working age (16–64 years old), representing 6.2% of the total working-age population. Regarding the collective, it stands out that, among other aspects regarding them, their activity rate is 34.0%, which is more than 43 points below that of the people without disabilities. Furthermore, their employment rate is 25.9% (a difference of 41 points above that of the people without disabilities). Moreover, their unemployment rate was 23.9%, while the indicator among the people without disabilities is 13.9%. These data not only show that the labor activity among PWDs is significantly lower than that among the people without disabilities but also that, in line with it, their entrepreneurship is lower than that among the people without disabilities. Furthermore, data of Observatorio sobre Discapacidad y Mercado de Trabajo en España (ODISMET) (ODISMET, 2016, 2018, 2020) indicate that there are 54,900 self-employed PWDs, of whom 28.6% are employers, 67.9% are entrepreneurs without employees, and 3.5% have other professional situations, so that the registered self-employment rates were 3.2, 7.6, and 0.4%, respectively, being those figures similar to those among the collective of the population without disabilities (4.8, 10.4, and 0.4%).

The entrepreneurial capacity of the collective of PWDs can be highly valuable for the society for two reasons: on one hand, because it involves the recognition of the rights of such collective and the increase of the possibilities that enable PWDs to benefit both from social inclusion and the consequent rehabilitative, therapeutic, and dignifying effect that it has on them, and on the other hand, because it is also likely to generate benefits for the community as the obstacles that hinder such social inclusion are removed and opportunities for vulnerable collectives are created (Troncoso, 2014).

Even if it is hard to overcome the difficulty of measuring social value in economic terms, we have chosen as the basis of our research the study of the concept of “shared value” as suggested by Porter and Kramer (2011) or Bonini and Emerson (2005), and according to them, what defines this value is not only the competitiveness of an organization but also those policies and operational practices that improve the social and economic conditions of the community. This latter condition that suggested the meaning of the concept of “shared value” is relevant to develop policies that promote the integration of PWDs into the labor market. To that extent, instruments such as the social return on investment (SROI) provide a framework for measuring this concept of value, which is expressed in terms of money as a common unit. Moreover, social and environmental value is included in such an approach thanks to the use of market proxies (Nicholls et al., 2012; Mook et al., 2015). However, even if it would be wrong not to recognize the importance of quantifying actions relevant to the people or organizations experiencing it or contributing to the concept of social value, to consider those actions the meaning of that concept may go beyond economics (Austin et al., 2006; Vásquez and Dávila, 2008; Wood and Leighton, 2010).

In line with this understanding of “shared value,” the entrepreneurship of vulnerable collectives, such as the PWDs, apart from having economic value as an entrepreneurial project, also has social repercussions and social objectives and, therefore, is likely to create social value. A large part of the entrepreneurial activity carried out by PWDs is focused on activities related to the third sector. The data published by the Observatorio Estatal de la Discapacidad (2019) are significant regarding the contribution of disability-related entities to the generation of social value: 10,500 organizations, of which 73.3% are more than 20 years old and represent 36% of the social action third sector, in which—according to estimates made in 2013 −5,181 million euros were earned and 4,894 million euros were spent alone in that year, 52% of the entities recorded a positive balance sheet, 28% recorded a compensated one, and, last but not least, 92% generated the funds for their own self-financing, what reveals excellent economic management in the sector.

On a microscale, these are companies whose aim is to solve social and environmental problems through the market (Beaumont, 2016; Javier and Zamudio, 2018). In short, social value is created as a contribution to collective or individual well-being so that society is improved by seeking a response to unsatisfied needs of a different nature (Austin et al., 2006). Therefore, the process of entrepreneurship “involves a balance of social behavior and economic behavior, which, in turn, enables the generation of both social and economic value” (Chell, 2007: 17).

Following the experiences of previous research studies, the identification of what favors and what hinders PWDs from becoming entrepreneurs has become the central research question. This study approaches such issue in a different way going beyond the purely sociodemographic and economic aspects to explore the knowledge, skills, and abilities that are likely to activate entrepreneurship (Olaz, 2011). The objective approach to these competencies—whether individual or social—is a matter of utmost importance. Thus, the creation of instruments that contribute to such an approach acquires a major added value. This study contributes to this direction in the first place, because its focus is on the analysis of the competencies that are self-attributed and may encourage the collective of PWDs, whose disabilities may be physical, organic, or sensory, to undertake an entrepreneurial activity. Moreover, the analysis also involves the relationship between entrepreneurship and positive conditions for the autonomy of that collective, which, if met, will also create “social value” in the sense indicated above.

When tracing the competency profile of the entrepreneurial condition of PWDs, the following question arises: Is it possible to speak of differences in entrepreneurship according to the condition of belonging to that collective? Some sociodemographic peculiarities in the entrepreneurship of PWD have been identified in the context of the project called Global Entrepreneurship Monitor (GEM) and through studies based on the data analysis of entrepreneurship in the world (Moreno et al., 2016; Barragán et al., 2019). These differences refer to elements such as age, educational level, and stereotypes/pre-judices regarding their capacity, or motivation to create a business. Therefore, it has been considered that, concerning the activity, those factors are limiting (Mercado et al., 2013; Moreno and Tejada, 2016). However, in-depth analysis in the aspect of the competencies is missing, even if the entrepreneurial initiative is subject to such analysis.

Also, according to further research studies on that question, there is a lack of entrepreneurial activity among PWDs due to the absence or scarcity of specific measures to promote their entrepreneurship and poor involvement of public authorities or, in other words, a lack of funding to support the collective so that, as a professional alternative, they can develop their business idea. Meanwhile, some authors identify other aspects, such as social prejudices related to their labor potential, difficulties of accessibility, and the development of passive policies, which encourage PWDs to look for a labor alternative (Moreno, 2016). However, there are not enough research studies on analyzing how PWDs perceive themselves concerning entrepreneurship or precisely identified what attributed competencies are needed to carry out an initiative of this type.

In relation to the above, it could be expected that there is an obvious link between shared value and social value in relation to the group we are looking into. In this regard, policies aimed at improving the competitiveness of this collective result in the generation of social value from their full integration into the labor market, with the social and economic benefits resulting from that integration.

In conclusion, the objective of this research study is to explore the competencies that generate social value among entrepreneurial PWDs.

## Literature Review From Competencies to Autonomy and the Creation of Social Value

Subsequently, we went through a review of the literature that associates entrepreneurship, skills, and autonomy in PWDs.

### Entrepreneurship, Competencies, and Disability

The sociological perspective has not been one of the most widely used in research on entrepreneurship, which has led to an insufficient interpretation of the topic in an “asocial” and “timeless” way (Pereira, 2007: 19), hence the relevance of research studies where this point of view is adopted. According to this perspective, entrepreneurship is considered as a manifestation not only of social change but also of the integration of economic and social forces. Therefore, promoting this activity in vulnerable groups is of great social value. This approach has Weberian roots but has not been sufficiently explored. From the 1980s onward, a series of academic studies on the subject emerged from very different perspectives including the exploration of social and geographical mobility and its influence on the propensity for entrepreneurship (Hagen, 1968), the determination of contextual factors such as the existence of social networks and resources of diverse nature that condition entrepreneurship (Gibb and Ritchie, 1982; Ajzen, 1988; Burt, 2000), or the way how a combination of factors that operate in the long term relativize the entrepreneurial determination (Giraudeau, 2007). The competency approach combines a sociological and psychological perspective, as competencies are acquired in a social context that conditions their acquisition and possibilities of development.

Competencies are the formal translation of the skills required and self-demanded for the development of the entrepreneurial project. They are a construct that makes it possible to identify the behaviors responsible for performance according to dimensions such as knowledge, skills, and abilities (Olaz, 2011; Olaz and Ortiz, 2016a).

Even if the link between entrepreneurship and competencies has been already mentioned in previous research studies on that field, it developed an approach to such relation generally basing on the exploration of the psychological characteristics of the entrepreneur and, in particular, on the analysis of some aspects related to leadership skills and risk management. In this vein, research studies such as that of Raičević et al. (2007) identify strategic competencies for entrepreneurship and the entrepreneurial activity itself. Among the strategic attitudes, the authors include the willingness to show initiative, the positive attitude to change and innovate, and the willingness to identify areas where entrepreneurial skills can be evidenced.

Other research studies focus on perceptions as determining factors when undertaking an entrepreneurial activity, such as the perception of risk related to entrepreneurship (Brindley, 2005), not to forget those factors whose interest lays on the perception of attitudinal strengths, such as self-efficacy, control, and the need for achievement, all of which are attitudes that are very present in people who have had successful entrepreneurial experiences (Rábago et al., 2004).

In academic literature, there have been abundant research studies on motivation since 1943, as Maslow (1943) indicated that the motive that encourages to undertake is the search for the satisfaction of many needs. In other words, motivation arises from a state of imbalance or tension in the search to overcome a set of needs represented in the set of capabilities of the already well-known Maslow's pyramid. These references suggest the importance of motivation as a psychosocial category without relating it yet to the concept of competencies.

In the 1990s, interest in the study of competencies became active in two main areas: on the one hand, in an area that is related to the processes associated with professional qualification and the labor market, in which the research works of Wolf (1994), Civelli (1997), Beret and Dupray (1998), Guerrero (1999), and Dodd et al. (2002) stand out; on the other hand, in an area which is more focused on management, that is represented by those of Merle (1997), Velde (1999), and Mulcahy (2000).

More recently, innovative approaches that went beyond the traditional training, entrepreneurial scheme, emerged as research focused on two specific fields: With regard to the first field, approaches referred to knowledge such as several research works that focused on models that were developed both in Bereiter's model about knowledge construction (Bereiter, 2002) and in innovative communities where learning is central (Paavola et al., 2004). In this line, it is also worth mentioning the suggestion of Billett about knowledge construction in the workplace (Billett, 1994).

As for the second field, other approaches referred to the process in which the concept of socio-emotional competencies relegates that of competency performance to a second status. In the same vein, the notion of performance seems to have been replaced by a different notion, which is related to a set of aspects concerning the potential and personal qualities of an individual as studied by the (Hay Group, McClelland Center for Research and Innovation, 2005), which suggested in its work the different performance domains (Burckle, 2000; Humphrey et al., 2001 and Sevinc, 2001; Stagg and Gunter, 2002; Nel and De Villiers, 2004).

Therefore, more and more studies emerge that, to some extent, show sensitivity toward the psychosocial component of competencies and motivation as a key competence. Nevertheless, the suggested definition concerning entrepreneurial processes is not clear and, from a competency perspective, such a definition is even more imprecise; hence, the singularity of the research study on entrepreneurship in PWDs from a competency perspective developed in Olaz and Ortiz (2016b, 2017, 2018) and Ortiz and Olaz (2019a,b, 2020).

Among the constraints to entrepreneurship in PWDs indicated in the research study, there are personal or psychological factors (Ortiz and Olaz, 2020) that are derived from the disability itself and hinder learning and developing social skills to a full extent. This is worsened by the lack of empathy of society toward these potential entrepreneurs, as a result of which they suffer from fear of failure and lack of self-esteem, self-confidence, and motivation. This reveals the importance of all aspects related to the competence framework. Thus, they should be prioritized over less influential factors such as political, institutional, legal, cultural, or environmental ones so that the understanding of this subject in question is enhanced.

Moreover, a research study that applies to entrepreneurship, the Theory of Expectations (Barba-Sánchez and Atienza-Sahuquillo, 2017), indicates that entrepreneurship in PWDs stems from an increase of the confidence in the competencies and skills needed to carry out the business project.

In the same vein, Caldwell et al. (2016) and Dhar and Farzana (2017) consider that self-confidence is the main premise of entrepreneurship among PWDs. According to these authors, the social and economic relevance of the contribution to the creation of autonomous identity is from where PWDs can get the main motivation to become entrepreneurs and to overcome obstacles such as lack of access to information and training or those related to mobility and functioning. Empirical studies show that competencies and successful entrepreneurial experiences are implemented by PWDs in entrepreneurship (Obschonka et al., 2017). In addition, empirical research study finds evidence of the application of the competencies learned in professional and life history during the undertaking of an entrepreneurial project (Sánchez-García and Suárez, 2017) and using career self-management competencies, especially, the eagerness to learn and self-education (Alvarado et al., 2020). In that research study, it is concluded that the competencies that explain entrepreneurial success in PWDs include specific professional competencies, experience in the sector, ability to learn new things in business practice, ability to recognize opportunities, and socio-emotional competencies, such as being patient and knowing how to listen to people, being able to deal with uncertainties in the case of an innovative business idea, developing emotional and communication skills, and knowing how to seek and accept support.

Having reviewed the literature on entrepreneurship among PWDs, we have learned that it is not only difficult to find contrasted research studies on entrepreneurship from a competency-based perspective but, even more, to find those whose central focus is on disability. It is therefore interesting to discuss competencies and, in particular, self-attribution of competencies, especially as they are related issues. Even though they have not been addressed, they are a factor to be considered concerning the entrepreneurship of PWDs and, thus, support the working hypothesis H1.

### Entrepreneurship and Autonomy Among PWDs

Analyzing the motivations that encourage an individual to become an entrepreneur involves talking about what conditions his behavior (namely, his personal and social circumstances, and factors of his environment). According to Westhead (Westhead et al., 2011), certain personality traits, such as the need for achievement, as well as for autonomy, the propensity to take risks, creativity, or having self-confidence, direct an individual toward entrepreneurship (Kuratko and Hodgetts, 2007; Chell, 2008; Dollinger, 2008; Price, 2013; Rampton, 2014). Although these personality traits are independent of the disability status, according to empirical studies, the collective of PWDs manifests a lower confidence and risk-taking capacity. Among the reasons why in that collective such behavior is developed, there is not only the internalization of social prejudices but also the low capacity of PWDs to create business networks. Even if networking is important for the creation and development of entrepreneurial projects, as well as for the expansion of business relationships, in this regard, PWDs face more obstacles than other collectives (Dhar and Farzana, 2017). Nonetheless, entrepreneurship is an interesting alternative for the collective of PWDs as it may empower the individual (Balcázar et al., 2014).

The trinomial consisting of the three factors, namely, empowerment, autonomy, and self-fulfillment, is analyzed by Shogren and Shaw in their research study on PWDs (Shogren and Shaw, 2016). In such a research study, they examined to which extent, out of four core characteristics of self-determination (autonomy, self-regulation, psychological development, and self-fulfillment), the three factors included in the trinomial predicted the quality of life of adults belonging to the collective of PWDs. Their research study was based on the definition of autonomy as the degree to which a person acts following his or her preferences, interests, and abilities, instead of being bound to undue external influences (Wehmeyer and Palmer, 2003). The authors of the research study identified that there was a relationship between autonomy, psychological empowerment, self-fulfillment, and employment in the collective of PWDs, which is an interesting issue, especially as they compared that collective and the general population and evidenced inequalities in their integration in the labor market. This suggests that the personal transformation that the individual inevitably needs to undergo to enter the labor market or while developing a professional career confronts this collective with many obstacles (Schur et al., 2017), being those obstacles linked to autonomy and poor skills for getting a job (Lindsay et al., 2015).

Entrepreneurship is an important means of gaining autonomy and thus overcoming obstacles and implementing skills that lead an individual to get employed (Nevala et al., 2015). In this vein, achieving a certain degree of independence is, along with entrepreneurship and the recognition of entrepreneurial opportunities, one of the main motives of undertaking an entrepreneurial activity (Lindsay et al., 2015; Moulton and Scott, 2016). Moreover, launching an own enterprise is a way of dealing with inequalities in the labor market, which is the reason why this activity has such a high social value (Alvarado et al., 2020).

The concept of autonomy referred to here is related to two aspects: first, the freedom of action of an individual—in the sense defined by Wehmeyer and Palmer (2003)—and second, the ability to achieve independence from the family nucleus, so that, on one hand, there is a greater need for self-financing, and on the other, there are also more possibilities to do so.

In this regard, it is necessary to consider the importance of the support of the family of PWDs in their development. In this case, we refer to the support of the primary family, which plays a role in the economic activity of the collective of PWDs and, in particular, in the entrepreneurial possibility as great as in the no less important resilience of entrepreneurs with disabilities (EWDs) (Knox, 1993; Porcelli et al., 2014; Ge et al., 2021). However, the primary family must necessarily facilitate the autonomous development among the collective of the PWDs and avoid overprotective dynamics that weaken the implementation of skills and competencies (Mendoza and Roldan, 2019). In this vein, there are research studies on the subject that consider the participation of the family in the labor market insertion process unnecessary, following the belief that the concept of normalization implies that the family should not be involved and should give way to the independence and autonomy competencies of PWDs, given that these competencies are aimed at adults (Valls et al., 2004). In this sense, the family can become an obstacle to the development of an autonomous professional project. The idea is that the individual enters an environment—such as that of the company—that is not based on the logic of protection and care but on that of profitability and competition. So, the family is a means of resocializing the collective of PWDs giving the individuals of such collective decision-making capacity and responsibilities instead of too much protection and care (López and Seco, 2005).

In short, the review of the literature on the subject has allowed establishing the analysis model and the working hypotheses. According to the empirical research studies, a relationship can be determined between competence self-attribution and entrepreneurship in the collective of PWDs (Nevala et al., 2015; Olaz and Ortiz, 2016a,b, 2017, 2018; Barba-Sánchez and Atienza-Sahuquillo, 2017; Obschonka et al., 2017; Ortiz and Olaz, 2019a,b, 2020; Alvarado et al., 2020). In this new research study, the competency attribution profile among entrepreneurial PWDs has been traced using the Emotional Competence Inventory (ECI)^1^. Furthermore, a total of 18 competencies^2^ have been classified into four dimensions: personal self-knowledge; self-management; social awareness; and relationship management.

**Figure 1:**
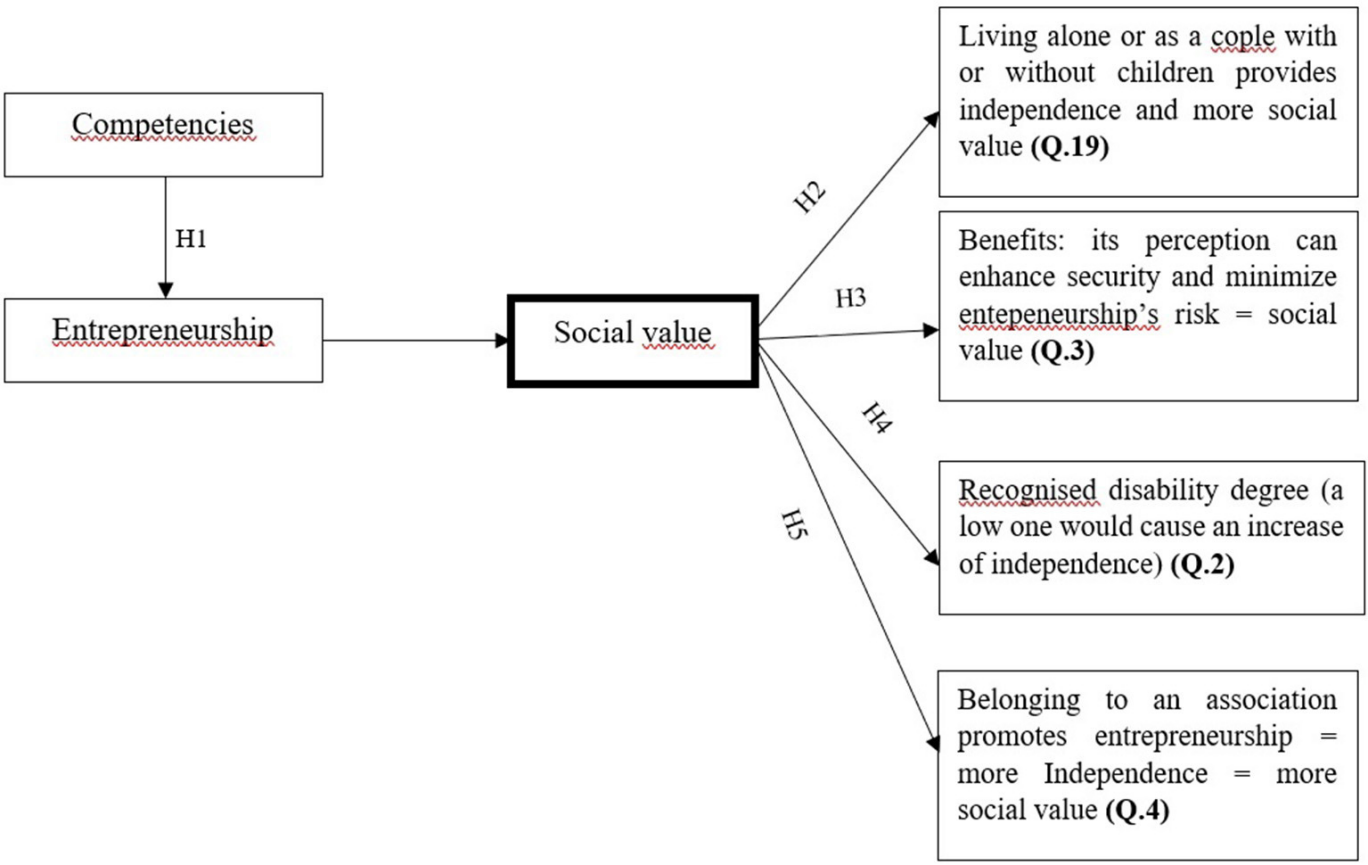
Summarized approach to the analysis of entrepreneurship social value of PWDs. Source: Own elaboration.

Regarding the link between PWDs entrepreneurship and social value, correlations between entrepreneurship and autonomy are one of the main findings of empirical research studies. In the resulting literature, it has been demonstrated that such correlations are liable to prove the creation of social value in this activity (Jones and Latreille, 2011; Toboso and Rogero, 2012; Balcázar et al., 2014; Lindsay et al., 2015; Moulton and Scott, 2016; Barba-Sánchez et al., 2019, 2021; Moreno, 2019). According to this evidence, the focus of this research study is on the autonomy of PWDs. The analysis of such autonomy, both economic and from the family, is based on issues related to the cohabitation form and to the benefit perception (Q. 19 and 2 of the questionnaire and Appendix). The analysis includes the recognized disability degree considering it as a moderating factor of autonomy (Q. 3). Another factor is the participation in a disability association, for which the existence of a social network is implied, likely to influence the propensity to develop an enterprise. According to such an approach, the mentioned analysis is graphically represented in the following figure:

Taking the model into consideration, based on the literature review that links entrepreneurial competencies in PWDs and social value (Akinyemi, 2016; Barba-Sánchez and Atienza-Sahuquillo, 2017; Barba-Sánchez et al., 2019, 2021), the hypotheses explored in this article are the following (Figure 1):

H1. Competencies are positively related to entrepreneurship, as among entrepreneurial people the self-assessment of their competencies is better than among those who neither have undertaken an entrepreneurial activity nor are likely to undertake one.
H2. Entrepreneurship is significantly related to the capacity of individual autonomy of living alone or as a couple with or without children.
H3. Receiving benefits is positively related to entrepreneurship, given that it can reduce the perception of risk, which is one of the main problems for entrepreneurship. Consequently, the generation of social value depends on it.
H4. The recognized disability degree moderates the possibility of entrepreneurship as follows: the higher the degree of disability, the lower the possibility of entrepreneurship. Consequently, it implies that social value is reduced.
H5. Belonging to an association is positively related to entrepreneurship causing a greater degree of independence so that the consequence would be the desired one.

## Materials and Methods

This research study has been developed according to the results of a nationwide survey carried out between November and December 2018. Its target group consists of people with some type of physical, organic, and sensory disability. The data collection tool, which is based on a questionnaire, has been designed considering its form and the singularity of such target group when accessing information. The collaboration of the main national disability support associations (ONCE, FAMDIF-COCEMFE) was decisive for the design of the sample. The total population of the indicated type is 986,600 PWDs (ODISMET, 2016, 2018, 2020), out of which 224 valid responses were obtained, which represent a sampling error of 6.7% and a confidence level of 95.5% (*p* = *q* = 0.05). As for the rest of the technical characteristics of the sample, the target population is between 18 and 64 years old residents in Spain with physical, sensory, and organic disabilities. The survey was based on a face-to-face questionnaire that was validated in terms of navigability by the Diversity and Volunteering Service of the University of Murcia, as well as by the technical services of Organización Nacional de Ciegos Españoles (ONCE). Furthermore, it was carried out between November and December 2018 and consists of 22 questions, structured in four thematic blocks (Annex 1).

As for the analysis, it was based on two statistical operations: the Mann–Whitney *U* non-parametric test for two-level factors and a bimodal logistic regression. The first of these operations was used to analyze competency factors self-attributed by PWDs (Q.14, Appendix 1) and their relationship with entrepreneurship (H1), while H2, H3, H4, and H5 have been analyzed using a logistic regression expressed through the following formulation:

### Undertaking of an Entrepreneurial Activity ~ P19 + P2 + P3 + P4

In order to achieve a more appropriate operationalization, we have proceeded to initially recode the variable entrepreneurship (Q. 5) so that a new variable (starting up a business) was created, which reveal the following data: Yes, they start up a business (94 PWDs); no, they do not start up any business (88 PWDs); and na (42 PWDs).

As for the treatment of the variables, the variable “starting up a business” resulting from the recoding of the variable “entrepreneurship” (Q. 5) was taken as the dependent variable, while the independent variables were “cohabitation form” (Q. 19); “benefits” (Q. 3); “ disability degree” (Q. 2), and “membership in an association” (Q. 4).

## Results

### Entrepreneurship in PWDs and Self-Attribution of Competencies

Taking into consideration the conducted analysis on the self-attributed competencies depending on whether or not PWDs have been entrepreneurs (Table 1), the average values indicate that the entrepreneurial PWDs attribute a greater capacity to themselves than the non-entrepreneurial ones, except in competencies 1 (“emotional awareness”: 4.38 entrepreneurs, compared to 4.42 non-entrepreneurs), 5 (“transparency”: 4.35 entrepreneurs, compared to 4.36 non-entrepreneurs), and 6 (“adaptability”: 4.28 entrepreneurs and non-entrepreneurs). The exceptions point to the case when the evaluation given to certain competencies among non-entrepreneurial PWDs is equal or slightly higher than that among PWDs who have started up a business. Such exceptions are located in the dimension “personal self-knowledge” (competence 1 – emotional awareness) and “self-management” (competencies 5 – transparency and 6 – adaptability). On the contrary, competencies that belong to the dimensions “social awareness” and “relationship management” receive a higher rate according to the self-assessment of entrepreneurial PWDs than when evaluated by non-entrepreneurial PWDs. In the latter two dimensions, there is a statistically significant relation, which is present in competencies 9, 12, and 15 (optimism; service orientation; and catalyzing change). Meanwhile, in the “self-management” dimensions, significant is the relation in competence four (emotional self-management). According to the data, it may be noted that entrepreneurial PWDs attribute to themselves a higher competency value than non-entrepreneurial PWDs, which confirms the hypothesis (H1 entrepreneurial PWDs—the self-assessment of their competencies is better than among non-entrepreneurial PWDs).

**Table 1:** Descriptive data according to entrepreneurship and competencies of PWDs.

	**Competency**	**Entrepreneur**	***N***	**Mín**	**Max**	**Mean**	**Median**	**%1**	**%2**	**%3**	**%4**	**%5**	**SD**	**W**	***p*-valor**
Dimension 1. Personal self-knowledge	1. Emotional awareness	Sí	116	2	5	4.38	5.00	0.00	1.72	13.80	29.30	55.20	0.79	3282	0.621
		No	59	2	5	4.42	5.00	0.00	3.39	10.20	27.10	59.30	0.81		
	2. Self-assessment	Sí	116	2	5	4.55	5.00	0.00	0.86	6.03	30.20	62.90	0.65	3282	0.733
		No	60	2	5	4.52	5.00	0.00	1.67	5.00	33.30	60.00	0.68		
	3. Self-confidence	Sí	115	2	5	4.30	4.00	0.00	2.61	14.80	33.00	49.60	0.82	3550	0.733
		No	60	2	5	4.18	5.00	0.00	8.33	16.70	23.30	51.70	1.00		
Dimension 2. Self-management	4. Self-control	Sí	116	1	5	4.05	4.00	0.86	4.31	16.40	45.70	32.80	0.86	4008.5	0.0489^*^
		No	59	2	5	3.83	4.00	0.00	1.69	37.30	37.30	23.70	0.81		
	5. Transparency	Sí	116	2	5	4.35	4.00	0.00	0.86	11.20	39.70	48.30	0.71	3317.5	0.872
		No	58	3	5	4.36	5.00	0.00	0.00	15.50	32.80	51.70	0.74		
	6. Adaptability	Sí	116	1	5	4.28	5.00	0.86	2.59	17.20	26.70	52.60	0.90	3548	0.818
		No	60	2	5	4.28	4.00	0.00	1.67	16.70	33.30	48.30	0.80		
	7. Achievement	Sí	115	1	5	3.97	4.00	3.48	6.09	20.90	29.60	40.00	1.08	3402	0.976
		No	59	1	5	3.90	4.00	5.08	10.20	20.30	18.60	45.80	1.24		
	8. Initiative	Sí	114	1	5	4.17	4.00	0.88	2.63	18.40	35.10	43.00	0.88	3527.5	0.717
		No	60	1	5	4.10	4.00	1.67	1.67	25.00	28.30	43.30	0.95		
	9. Optimism	Sí	115	1	5	4.33	4.00	0.87	0.87	11.30	38.30	48.70	0.78	4209	0.01^*^
		No	60	1	5	3.97	4.00	3.33	1.67	21.70	41.70	31.70	0.96		
Dimension 3. Social awareness	10. Empathy	Sí	116	2	5	4.35	5.00	0.00	2.59	12.90	31.00	53.40	0.80	3735	0.383
		No	60	1	5	4.23	4.00	1.67	3.33	10.00	40.00	45.00	0.89		
	11. Organizational competency	Sí	114	1	5	4.18	4.00	2.63	0.00	17.50	36.00	43.90	0.91	3692.5	0.258
		No	59	1	5	4.05	4.00	1.69	3.39	15.30	47.50	32.20	0.88		
	12. Service orientation	Sí	115	3	5	4.31	4.00	0.00	0.00	11.30	46.10	42.60	0.67	3458.5	0.0459^*^
		No	51	1	5	4.00	4.00	1.96	1.96	23.50	39.20	33.30	0.92		
Dimension 4. Relationship management	13. Developing people	Sí	115	2	5	4.01	4.00	0.00	1.74	26.10	41.70	30.40	0.80	3714.5	0.195
		No	58	1	5	3.74	4.00	3.45	10.30	17.20	46.60	22.40	1.04		
	14. Leadership	Sí	113	1	5	3.98	4.00	1.77	1.77	23.00	43.40	30.10	0.88	3456.5	0.537
		No	58	1	5	3.83	4.00	3.45	8.62	22.40	32.80	32.80	1.09		
	15. Catalyzing change	Sí	109	1	5	4.24	4.00	1.83	0.92	15.60	34.90	46.80	0.88	3735.5	0.00303^*^
		No	54	1	5	3.65	4.00	9.26	5.56	27.80	25.90	31.50	1.25		
	16. Influence	Sí	113	2	5	4.19	4.00	0.00	2.65	19.50	33.60	44.20	0.84	3541.5	0.258
		No	57	1	5	3.98	4.00	5.26	1.75	15.80	43.90	33.30	1.03		
	17. Conflicts management	Sí	114	2	5	4.40	5.00	0.00	0.88	10.50	36.00	52.60	0.71	3533.5	0.214
		No	56	1	5	4.16	4.00	3.57	1.79	16.10	32.10	46.40	1.01		
	18. Teamwork	Sí	113	3	5	4.62	5.00	0.00	0.00	7.08	23.90	69.00	0.62	3411.5	0.593
		No	58	1	5	4.45	5.00	3.45	1.72	8.62	19.00	67.20	0.98		

*The columns headed by percentages contain the percentage of people who responded in terms of the percentages heading each of the columns. Following the Likert scale, the percentages given in their responses range from 1% (“not at all competent”) to 5% (“very competent”)*.

*Source: PWD survey. Project “Disability and entrepreneurship. Competency analysis” CSO2016-75818–R (AEI/FEDER, UE)*.

* *means the result is statistically significant (<0.05)*.

### Entrepreneurship, Autonomy, and Social Value

As established in the research model (Table 1), the relation between entrepreneurship and autonomy (H2) is analyzed below. The resulting data indicate that 53.4% of the PWDs launch a business out of necessity, while the rest do it out of the opportunity. Furthermore, autonomy is one of the fundamental motives for PWDs to start up a business that 33.9% of PWDs start up a business to increase income, 30.5% to achieve greater personal independence, 27.1% to overcome a personal challenge, and the remaining percentage does not answer the question. Those are their main reasons, which are personal rather than economic, and prioritize economic independence and the self-satisfaction derived from autonomy.

As a result of the regression analysis (RA) (Table 2), it has been initially established that the significant values would refer to life in a couple without children (*p* 0.0421 < 0.05). No statistical significance was found in cases as respondents neither lived with family members under their charge nor had any other living situation.

**Table 2:** Statistical summary of logistic regression n.1 with results for all variables.

	**Estimate std**.	**Error**	***z* value**	**Pr(>|z|)**
(Intercept)	1.09528	0.58934	1.858	0.0631.
P19V I live in company without children	1.02819	0.50580	2.033	0.0421^*^
P19V I live in company with children	0.65455	0.53173	1.231	0.2183
P2 from 50–64%	−0.01409	0.66411	−0.021	0.9831
P2 from 65–74%	−1.11350	0.64996	−1.713	0.0867.
P275% and more	−0.86794	0.56222	−1.544	0.1226
P2 Na	15.87135	1661.43837	0.010	0.9924
P3 No	−1.04478	0.47791	−2.186	0.0288^*^
P3 Na	−17.20240	2399.54482	−0.007	0.9943
P4 No	0.30436	0.47220	0.645	0.5192

<0.05“^*^”. AIC = 170.66.

*Source: PWD survey. Project “Disability and entrepreneurship. Competency analysis” CSO2016-75818-R (AEI/FEDER, EU)*.

Concerning the relation between entrepreneurship and economic autonomy, where entrepreneurship has been identified through the reception of disability benefits, significant is “not receiving any type of benefit” (*p* 0.0288< 0.05). In other words, among the entrepreneurial PWDs, the reception of disability benefits is no motivational element toward entrepreneurship.

While there are some entrepreneurial capacity moderating factors, such as the recognized disability percentage, which is situated in the medium–high interval, namely, between 65 and 74%, which are practically significant, there are also other factors, such as belonging to an association, which are found not to be significantly related to the entrepreneurship of PWDs.

Regarding these results, the model has been optimized aiming at improving the final Akaike information criterion (AIC), thus obtaining the variables shown in Table 3. According to the logistic regression, it is confirmed that the living situation “I live together without children” is a statistically significant variable, which demonstrates that such a variable is consistent in predicting the entrepreneurship of PWDs.

**Table 3:** Optimized logistic regression n.2.

	**Estimate std**.	**Error**	***z* value**	**Pr(>|z|)**
Intercept	0.6270	0.4577	1.370	0.1707
P19V I live in company, without children	1.0237	0.4761	2.150	0.0315^*^
P19V I live in company, with children	0.7763	0.5047	1.538	0.1241
P3 No	−0.6947	0.4327	−1.605	0.1084
P3 Na	−16.9693	1455.3976	−0.012	0.9907

<0.05“^*^”. AIC = 168.82.

*Source: PWD survey. Project “Disability and entrepreneurship. Competence analysis” CSO2016-75818-R (AEI/FEDER, EU)*.

Table 4 shows the ratios and CIs of the variables considered in the adjusted model, hence the optimization of the initial AIC. Furthermore, odds ratios above 1 for the variables relating to cohabitation (living with others with or without children) indicate that the predictors influence entrepreneurship. In contrast, the variable “not receiving disability benefits” does not influence the model, as its odds ratios below 1 show the following.

**Table 4:** Coefficients and CIs.

	**Odds ratio**	**2.5 %**	**97.5 %**
(Intercept)	1.87	0.78	4.8e+00
P19 I live in company, without children	2.78	1.10	7.2e+00
P19 I live in Company, with children	2.17	0.81	6.0e+00
P3 No	0.50	0.21	1.1e+00
P3 Na	0.00	NA	2.2e+121

*Source: PWD survey. Project “Disability and entrepreneurship. Competence analysis” CSO2016-75818-R (AEI/FEDER, EU)*.

The verification based on a statistic *X*^2^ (1) = 9.32 and a *p*-value = 0.05 demonstrates that the model with the predictor variables is significantly better than the model only with the constant.

Finally, the verification of multicollinearity, which is based on the variance inflation factors (VIFs), indicates that there is no multicollinearity in these predictor variables since the obtained values are below 10 (Table 5).

**Table 5:** Multicollinearity as a function of variance inflation factors (VIFs).

	**GVIF**	**Df**	**GVIF^(^1/(2*Df***
P19	1.101500	2	1.024463
P2	1.320900	4	1.035401
P3	1.181544	2	1.042587
P4	1.148053	1	1.071472

*Source: PWD survey. Project “Disability and entrepreneurship. Competence analysis” CSO2016-75818-R (AEI/FEDER, EU)*.

Having carried out a verification based on a statistic *X*^2^ (1) = 9.32 and a *p*-value = 0.05, we have obtained the demonstration that the model with the predictor variables is significantly better than the model only with the constant.

Finally, the verification of multicollinearity, which is based on the VIFs, indicates that there is no multicollinearity in these predictor variables since the obtained values are below 10 (Table 5).

According to the obtained results, on one hand, hypothesis H1 has been confirmed that a relation between entrepreneurial PWDs and the fact that their competency self-assessment is found to be positive. Also, hypothesis H2 has been corroborated, for there is a relation between autonomy and entrepreneurship of PWDs, especially when the living form is that of a couple without children.

On the other hand, according to the analytic model formulation, hypothesis H3 has not been confirmed, given that that entrepreneurship is significantly related to the fact of not receiving disability benefits but not to the fact of receiving them.

Concerning hypothesis H4, entrepreneurship is not related to all degrees of disability recognition but only certain ones. Thus, such a hypothesis has been only partially confirmed.

Finally, no verification of hypothesis H5, which establishes the relationship between association membership and entrepreneurship, has been carried out.

## Discussion and Conclusions

Understanding the social value as the result of an alignment between resources, processes, and policies, with the firm purpose of contributing to an improvement in the quality of life of a citizen, it is undeniable that there are many challenges, no less to say that there is an opportunity to achieve them.

Social policies should aim to increase the activity and employment rates of people with disabilities, as well as improving and dignifying their working conditions, while fighting discrimination. However, frequently the emphasis is placed in the forms of regular employment (in enterprises or public administrations with places reserved for PWDs) or protected employment (in dedicated centers) but not in self-employment where there is a lack of specificity toward activities intended for PWDs.

Even above and beyond business figures and economic performance, our society and, especially, companies and their great transformational power have increasingly begun to show greater concern to the social value that they are capable of generating and the social value they generate for the community (Harrison et al., 2020).

Neither is the relevant field of entrepreneurship nor that of people with some kind of disabilities alien to this reality given that such collective, even if it is most of the time “invisible,” can also contribute to the creation of social value. The socio-emotional competencies, functioning as a cornerstone between attitude and aptitude and, thus, catalyzing the process of empowerment, are important to dynamize entrepreneurship among PWDs not only at a personal level but also at a community level, for the destiny of that collective, which is to represent social value in our complex society.

In line with the available research studies (Nevala et al., 2015; Olaz and Ortiz, 2016a,b, 2017, 2017, Barba-Sánchez and Atienza-Sahuquillo, 2017; Obschonka et al., 2017; Olaz and Ortiz, 2018; Ortiz and Olaz, 2019a,b, 2020; Alvarado et al., 2020; Barba-Sánchez et al., 2021), the analysis of the results carried out in this research study has evidenced the relationship between assessment of their competencies and the fact of starting up an enterprise of PWDs.

There are two possible explanations for the phenomenon that people who have been entrepreneurs have a better competency self-assessment than those who have not: On one hand, the positive self-assessment of certain competencies is due to entrepreneurship, and on the other hand, entrepreneurial PWDs are identified by the positive self-assessment of those competencies. Even if it is impossible to choose one, whatever the explanation may be, such phenomenon is especially important in those competencies, which are directly related to activities that improve social behavior or social performance of PWDs, namely, the competencies belonging to the dimensions “social awareness” and “relationship management.” Therefore, it is interesting to note that, according to the entrepreneurial self-assessment of PWDs, such competencies are the most highly rated ones.

This first conclusion confirms those theories on the subject (Rábago et al., 2004; Raičević et al., 2007), which identify competencies, such as optimism, service orientation, and predisposition to change, that are strategic for entrepreneurship and the entrepreneurial activity itself. Not less to say that they have resulted statistically significant.

In the same vein as research studies on the topic (Jones and Latreille, 2011; Toboso and Rogero, 2012; Balcázar et al., 2014; Lindsay et al., 2015; Moulton and Scott, 2016; Dhar and Farzana, 2017; Moreno, 2019), we suggest that the link between autonomy and entrepreneurship of PWDs indicates how entrepreneurship and the involved effects contribute to the empowerment of the collective consequently generating social value in terms of quality of life and social inclusion improvement.

According to the analysis, results have identified a relationship between economic independence and entrepreneurship, which is based on the fact that starting up a business is positively linked to not receiving any benefits. The cause of such relationship may be the psychological empowerment of the entrepreneurial PWDs, which makes it worth engaging in activities that minimize the need for financial support, especially when that entrepreneur is familiarized with the fact that entrepreneurial actions and economic consequences are bound, what has been confirmed according to research studies on the issue (Shogren and Shaw, 2016). Furthermore, if entrepreneurship is carried out using financial solvency, but not public contribution, then it is likely to create social value (Barba-Sánchez et al., 2021).

The degree of recognized disability may be a limiting factor, yet according to the obtained data and the research studies carried out on the issue (Ortiz and Olaz, 2019a,b), it does not hinder entrepreneurship. As for membership of associations and its relation with entrepreneurship, while it may be true that entrepreneurship is enhanced thanks to the establishment of social support networks (SSNs), especially in the case of PWDs for whom such establishment is complicated (Dhar and Farzana, 2017), it is also true that membership of associations does not seem to be an indisputable predictor of the possibility of starting up any business activity.

This research study has focused on the attribution of competencies of PWDs concerning entrepreneurship and moderating variables such as autonomy. Consequently, a limitation of this investigation is that the exploration of the relationship between the inhibiting factors of assessment of competencies and entrepreneurial activity of PWDs has been out of the scope of this study and may be explored in future research studies. However, this research study has provided evidence on the social value of entrepreneurship in the collective of PWDs, which contributes to the development of a mature, fair, and equal society.

## Data Availability Statement

The raw data supporting the conclusions of this article will be made available by the authors, without undue reservation.

## Ethics Statement

The studies involving human participants were reviewed and approved by Comisión de Ética de Investigación de la Universidad de Murcia. The patients/participants provided their written informed consent to participate in this study.

## Author Contributions

POG and ÁO contributed to conception and design of the study and wrote sections of the manuscript. POG organized the database and the statistical analysis and wrote the first draft of the manuscript. Both authors contributed to manuscript revision, read, and approved the submitted version.

## Conflict of Interest

The authors declare that the research was conducted in the absence of any commercial or financial relationships that could be construed as a potential conflict of interest.

## Footnotes

^1^Hay Group's Emotional Competencial Intelligence (2005). Emotional Competence Inventory (ECI) Technical Manual, McClelland Center for Research and Innovation, prepared by WOLFF, S.B. DBA. Updated November 2005.

^2^Dimension 1. Personal self-knowledge (c1. Emotional conscience; c2. Autoevaluation; and c3. Self-belief); Dimension 2. Self-management (c4. Emotional self-control; c5. Transparency; c6. Adaptability; c7. Achievement; c8. Initiative; and c9. Optimism); Dimension 3. social awareness (c10. Empathy and c11. Organizational awareness) (c12. Orientación de servicio); and Dimension 4. Relationship management (c13. Developing people; c14. Inspiring leadership; c15. Catalyzation change; c16. Influence; c17. Conflicts management; and c18. Teamwork and collaboration).

## References

[B1] Ajzen I. (1988). Attitudes, Personality and Behaviour. Chicago: Dorsey.

[B2] Akinyemi E. O. (2016). Enterpreneurial empowerment of people with special needs in Ondo and Osun States, Nigeria. J. Arts Human. 5, 26–38. 10.18533/journal.v5i11.1013

[B3] Alvarado A. Suárez M. Sánchez M. F. (2020). Trayectorias emprendedoras en personas con discapacidad: características y condicionantes a través de estudios de caso. Psicoperspectivas 19, 1–12. 10.5027/psicoperspectivas-Vol19-Issue2-fulltext-1926

[B4] Austin J. E. Stevenson H. Wei-Skillern J. (2006). Social and commercial entrepreneurship: same, different, or both? Entrepreneursh. Theory Pract. 30, 1–22. 10.1111/j.1540-6520.2006.00107.x

[B5] Balcázar F. Kuchakb J. Dimpflc S. Sariepellac V. Alvarado F. (2014). An empowerment model of entrepreneurship for people with disabilities in the United States. Psychosoc. Interv. 23, 145–150. 10.1016/j.psi.2014.07.002

[B6] Barba-Sánchez V. Atienza-Sahuquillo C. (2017). Entrepreneurial motivation and self-employment: evidence from expectancy theory. Int. Entrepreneursh. Manage. J. 13, 1097–1115. 10.1007/s11365-017-0441-z

[B7] Barba-Sánchez V. Ortiz P. Olaz A. (2019). Entrepreneurship and disability: methodological aspects and measurament instrument. Journal Entrepreneurship Education, 22, 1–6.

[B8] Barba-Sánchez V. Salinero Y. Jiménez-Estévez P. (2021). Monetising the social value of inclusive entrepreneurship: the case of the Abono Café social economy enterprise. CIRIEC 101, 115–141. 10.7203/CIRIEC-E.101.18158

[B9] Barragán J. A. Moreno R. Tejada A. (2019). “El perfil del emprendedor con discapacidad en España en 2016. Análisis comparativo con los datos del informe GEM España (2016)” in Emprendimiento social, ocupación y discapacidad: II Congreso Nacional de Emprendimiento, Empleo y Discapacidad (Castelló de la Plana: Servei de Comunicació i Publicacions), 123–132.

[B10] Beaumont M. (2016). Gestión social: estrategia y creación de valor. Lima: Pontificia Universidad.

[B11] Bereiter C. (2002). Education and Mind in the Knowledge Age. Hillsdale: Lawrence Erlbaum.

[B12] Beret P. Dupray A. (1998). Valorizacion salarial de la formación profesional continua y producción de competencias por el sistema educativo: los casos de Francia y Alemania. Rev. Eur. Form. Prof. 14, 40–51.

[B13] Berger P. Luckmann T. (1966). The Social Construction of Reality: A Treatise in the Sociology of Knowledge. New York, NY: Doubleday.

[B14] Billett S. (1994). Situated learning: a workplace experience. Aust. J. Adult Commun. Educ. 4, 112–130.

[B15] Bonini S. Emerson J. (2005). Maximizing blended value–Building beyond the blended value map to sustainable investing, philanthropy and organizations. Available online at: http://community-wealth.org (accessed November 14, 2020).

[B16] Brindley C. (2005). Barriers to women achieving their entrepreneurial potential: women and risk. Int. J. Entrepre. Behav. Res. 11, 144–161. 10.1108/13552550510590554

[B17] Burckle M. (2000). ECI and MBTI. Hay/McBer Research Report.

[B18] Burt R. S. (2000). The network structure of social capital. Res. Organ. Behav. 22, 345–423. 10.1016/S0191-3085(00)22009-1

[B19] Caldwell K. Parker S. Renko M. (2016). Social entrepreneurs with disabilities: exploring motivational and attitudinal factors. Can. J. Disabil. Stud. 5, 211–244. 10.15353/cjds.v5i1.255

[B20] Chell E. (2007). Social enterprise and entrepreneurship. Int. Small Bus. J. 25, 5–26. 10.1177/0266242607071779

[B21] Chell E. (2008). The Entrepreneurial Personality: A Social Construction. New York, NY: Routledge/Taylor and Francis Group. 10.4324/9780203938638

[B22] Civelli F. (1997). New competences, new organizations in a developing world. Indust. Comm. Train. 29, 226–229. 10.1108/00197859710190742

[B23] Crespo E. Serrano A. (2013). Las paradojas de las políticas de empleo europeas: de la justicia a la terapia. Univ. Psychol. 12, 1111–1124. 10.11144/Javeriana.UPSY12-4.ppee

[B24] Dhar S. Farzana T. (2017). Barriers to entrepreneurship confronted by persons with disabilities: an exploratory study on entrepreneurs with disabilities in Bangladesh. Manage. Devel. 31, 73–96.

[B25] Dodd N. Brown F. W. Benham H. (2002). Learning to manage while learning about management: a transition to a competency-based management curriculum. J. Educ. Bus. 77, 189–192. 10.1080/08832320209599069

[B26] Dollinger M. (2008). Entrepreneurship: Strategies and Resources. Illinois: Irwin.

[B27] Ge Z. M. Chen R. X. Tang W. Z. Cong Y. (2021). Why strong employment support for persons with disabilities has not brought about positive outcomes? A qualitative study in mainland China. Child. Youth Serv. Rev. 121, 1–10. 10.1016/j.childyouth.2020.105839

[B28] Gibb A. Ritchie J. (1982). Understanding the process of starting small businesses. European Small Bus. J. 1, 26–46. 10.1177/026624268200100102

[B29] Giraudeau M. (2007). Le travail entrepreneurial, ou l'entrepreneur schumpetérien performé. Sociol. Trav. 49, 330–350. 10.1016/j.soctra.2007.06.025

[B30] Guerrero A. (1999). El enfoque de las competencias profesionales: una solución conflictiva a la relación entre formación y empleo. Rev. Complutense de Educ. 10, 335–360.

[B31] Hagen E. (1968). The Economics of Development. Irwin Press: Dorsey.

[B32] Harrison J. S. Phillips R. A. Freeman R. E. (2020). On the 2019 Business Roundtable “Statement on the Purpose of a Corporation”. J. Manage. 46, 1223–1237. 10.1177/0149206319892669

[B33] Hay Group McClelland Center for Research and Innovation. (2005). Updated November 2005 Emotional Competence Inventory (ECI), eds Steven B. Wolff D. B. A. Technical Manual.

[B34] Humphrey R. Sleeth R. Kellet J. (2001). Emotional Competence, Complex Task Choice, and Leadership Emergence. Unpublished Paper. Virginia Commonwealth University, School of Business.

[B35] INE (2020). El Empleo de las Personas con Discapacidad (EPD). Notas de prensa, 18 de diciembre de 2019. Available onlINEat: https://www.observatoriodeladiscapacidad.info/ (accessed May 24, 2021).

[B36] Javier E. Zamudio J. (2018). Comprensión del valor social creado por una empresa social bajo el enfoque de las capacidades: estudio de caso Shiwi. Master's Thesis. Pontificia Universidad Católica del Perú, Lima.

[B37] Jones M. Latreille P. (2011). Disability and self-employment: evidence for the UK. Appl. Econ. 43, 4161–4178. 10.1080/00036846.2010.489816

[B38] Knox M. T. R. (1993). Social networks and support mechanisms for people with mild intellectual disability in competitive employment. Int. J. Rehabil. Res. 16, 1–12. 10.1097/00004356-199303000-00001 8486438

[B39] Kuratko D. Hodgetts R. (2007). Entrepreneurship: Theory, Process, Practice. Ohio: Thomson South-Western.

[B40] Lindsay S. McDougall C. Menna-Dack D. Sanford R. Adams T. (2015). An ecological approach to understanding barriers to employment for youth with disabilities compared to their typically developing peers: views of youth, employers, and job counselors. Disabil. Rehabil. 37, 701–711. 10.3109/09638288.2014.939775 25014127

[B41] López C. M. Seco E. (2005). Discapacidad y empleo en España: su visibilidad. Innovar 15, 59–72.

[B42] Manzanera S. Ortiz P. (2017). “Discapacidad y su relación con el mercado de trabajo. Situación socio – laboral,” in Emprendimiento, empleo y discapacidad. Un diagnóstico, eds Ortiz P. Olaz A. (Aranzadi: Cizur Menor), 45–85.

[B43] Maslow A. (1943). A theory of human motivation. Psychol. Rev. 50, 370–396. 10.1037/h0054346

[B44] Mendoza M. G. Roldan S. (2019). Rol de familia en la integración de las personas con discapacidad física en los espacios de desempeño laboral. Revista Caribeña de Ciencias Sociales. Available online at: https://www.eumed.net/rev/caribe/2019/07/familia-personas-discapacidad.html (accessed November 14, 2020).

[B45] Mercado E. Aizpurúa E. García L. M. (2013). Avanzando hacia la igualdad de oportunidades en la inclusión socio - laboral de las personas con discapacidad. Cuadernos Trabajo Soc. 26, 95–104. 10.5209/rev_CUTS.2013.v26.n1.39571

[B46] Merle V. (1997). La evolución de los sistemas de validación y certificación: ¿Qué modelos son posibles y que desafíos afronta el país francés? Rev. Eur. Form. Prof. 12, 39–52.

[B47] Mook L. Maiorano J. Ryan S. Armstrong A. Quarter J. (2015). Turning social return on investment on its head. Nonprofit Manage. Leadersh. 26, 229–246. 10.1002/nml.21184

[B48] Moreno R. (2016). Inclusión, emprendimiento y empleo de las personas con discapacidad. Actualización y propuestas. Granada: La Ciudad Accesible.

[B49] Moreno R. (2019). El emprendimiento como alternativa al empleo ordinario en las personas con discapacidad. Revista de la Facultad de Derecho de México 69, 297–322. 10.22201/fder.24488933e.2019.273-1.68615

[B50] Moreno R. Blanco F. Barragán J. A. Polo C. Tejada A. López J.. (2016). “Análisis cuantitativo y cualitativo del emprendedor con discapacidad en España 2016,” in Inclusión, emprendimiento y empleo de las personas con discapacidad. Actualización y propuestas, eds Moreno R. (Granada: La Ciudad Accesible), 13–30.

[B51] Moreno R. and Tejada, A. (Coords.). (2016). Inclusión, emprendimiento y empleo de las personas con discapacidad. Actualización y propuestas. Granada: La Ciudad Accesible.

[B52] Moulton J. G. Scott J. C. (2016). Opportunity or necessity? Disaggregating self-employment and entry at older ages. Soc. Forces 94, 1539–1566. 10.1093/sf/sow026

[B53] Mulcahy D. (2000). Turning the Contradictions of Competence: competency- based training and beyond. J. Vocat. Educ. Train. 52, 259–280. 10.1080/13636820000200120

[B54] Nel H. De Villiers W. S. (2004). The relationship between emotional intelligence and job performance in a call centre environment. J. Indust. Psychol. 30, 75–81. 10.4102/sajip.v30i3.159

[B55] Nevala N. Pehkonen I. Koskela I. Ruusuvuori J. Anttila H. (2015). Workplace accommodation among persons with disabilities: a systematic review of its effectiveness and barriers or facilitators. J. Occup. Rehabil. 25, 432–448. 10.1007/s10926-014-9548-z

[B56] Nicholls J. Lawlor E. Neitzert E. Goodspeed T. (2012). A Guide to Social Return on Investment. Liverpool: SROI Network. Available online at: http://www.thesroinetwork.org/publications/doc_details/241-a-guide-to-social-return-on-investment-2012 (accessed May 24, 2021).

[B57] Obschonka M. Hakkarainen K. Lonka K. Salmela-Aro K. (2017). Entrepreneurship as a twenty-first century skill: entrepreneurial alertness and intention in the transition to adulthood. Small Bus. Econ. 48, 487–501. 10.1007/s11187-016-9798-6

[B58] Observatorio Estatal de la Discapacidad (2019). La sostenibilidad del Tercer Sector de la Discapacidad: Alternativas de financiación de los apoyos, servicios y estructuras. Available online at: https://www.observatoriodeladiscapacidad.info/ (accessed May 24, 2021).

[B59] ODISMET (2016, 2018, 2020). Fundación ONCE. Informe General. Principales resultados. Informe 5. Available online at: https://www.odismet.es/ (accessed May 24, 2021)

[B60] Olaz A. (2011). Una aproximación conceptual a la cualificación profesional desde una perspectiva competencial. Papers 96, 589–616. 10.5565/rev/papers/v96n2.154

[B61] Olaz A. Ortiz A. (Dirs.). (2017). Causas y factores del emprendimiento de personas con discapacidad. Un análisis competencial a través de la técnica de grupo nominal. Cizur Menor: Thomson Reuters – Aranzadi.

[B62] Olaz A. Ortiz A. (Dirs.). (2018). Discapacidad y emprendimiento. Dimensiones y contextos interpretativos en clave cualitativa. Cizur Menor (Navarra): Thomson Reuters – Aranzadi.

[B63] Olaz A. Ortiz P. (2016b). The competencial factor like an engine venture. Suma de Negocios 7, 2–8. 10.1016/j.sumneg.2015.12.001

[B64] Olaz A. Ortiz P. (Coords.). (2016a). Mujer y Emprendimiento desde una perspectiva competencial. Cizur Menor: Thomson Reuters – Aranzadi.

[B65] Ortiz P. Olaz A. (2019a). Entrepreneurship and disability. J. Entrepreneursh. Educ. 22:2.

[B66] Ortiz P. Olaz A. (2019b). Entrepreneurial activity dimensions a study in the persons with disability. Suma de Negocios, Konrad Lorenz 10, 1–8.

[B67] Ortiz P. Olaz A. (Dirs.). (2020). Discapacidad y emprendimiento. Notas metodoló a un proyecto de investigación. Valencia: Tirant Lo Blanch.

[B68] Paavola S. Lipponen L. Hakkarainen K. (2004). Models of innovative knowledge communities and three metaphors of learning. Rev. Educ. Res. 74, 557–576. 10.3102/00346543074004557

[B69] Pereira F. (2007). La evolución del espíritu empresarial como campo de conocimiento. Hacia una visión sistémica y humanista. Cuadernos Administr. 20, 11–36.

[B70] Porcelli P. Ungar M. Liebenberg L. Trépanier N. (2014). (Micro)mobility, disability and resilience: exploring well-being among youth with physical disabilities. Disabil. Soc. 29, 863–876. 10.1080/09687599.2014.902360

[B71] Porter M. Kramer M. (2011). Creating shared value. Harvard Bus. Rev. 89, 62–77. 10.1007/978-94-024-1144-7_16 10662001

[B72] Price J. (2013). Personality traits every successful entrepreneur has. Business Insider. Available online at: http://www.businessinsider.com/traits-of-successful-entrepreneurs-2013-2 (accessed May 24, 2021).

[B73] Rábago P. D'Annunzio M. C. Monserrat S. (2004). “Perfil de las mujeres emprendedoras exitosas de Argentina,” in II CIPEAL in Conferencia Internacional de Pesquisa em Emprendedorismo na America Latina. Disertation (Río de Janeiro).

[B74] Raičević S. Šćekić D. Vučurović V. Jaćimović Ž. (2007). Key competences for lifelong learning. Development of key competences in the Montenegrin education system. Torino: European Training Foundation.

[B75] Rampton J. (2014). Personality Traits of an Entrepreneur. Forbes. Available online at: https://www.forbes.com/sites/johnrampton/2014/04/14/5-personalitytraits-of-anentrepreneur/#2f9361a13bf4 (accessed December 5, 2014).

[B76] Sánchez-García M. F. Suárez M. (2017). Diseño y validación de un instrumento de evaluación de competencias para la gestión de la carrera emprendedora. Revista Iberoamericana de Diagnóstico y Evaluación Psicológica e Avaliação Psicológica 45, 109–123. 10.21865/RIDEP45.3.09

[B77] Schur L. Han K. Kim A. Ameri M. Blanck P. Kruse D. (2017). Disability at work: a look back and forward. J. Occup. Rehabil. 27, 482–497. 10.1007/s10926-017-9739-5 29110160

[B78] Sevinc L. (2001). The effect of emotional intelligence on career success: Research on the 1990 graduates of Business Administration Faculty of Istanbul University. Master's Thesis. Istanbul University, Istanbul.

[B79] Shogren K. A. Shaw L. A. (2016). The role of autonomy, self-realization, and psychological empowerment in predicting early adulthood outcomes for youth with disabilities. Remed. Special Educ. 37, 55–62. 10.1177/0741932515585003

[B80] Stagg G. Gunter D. (2002). Emotional Intelligence in the Fire Service. London: London Fire Brigade [Unpublished Paper].

[B81] Toboso M. Rogero J. (2012). “Diseño para todos” en la investigación social sobre personas con discapacidad. Revista Española de Investigaciones Sociológicas 140, 163–172. 10.5477/cis/reis.140.163

[B82] Troncoso N. G. (2014). Aporte de la empresa privada en la construcción de valor social y propuesta de modelo de medición de impacto. Master's Thesis. Universidad Técnica Federico Santa María, Santiago de Chile.

[B83] Valls M. J. Vilà M. Pallisera M. (2004). La inserción de las personas con discapacidad en el trabajo ordinario. El papel de la familia. Rev. Educ. 334, 99–117.

[B84] Vásquez A. G. Dávila M. T. (2008). Emprendimiento social. Rev. Literatura Estud. Gerenciales 24, 105–125. 10.1016/S0123-5923(08)70055-X

[B85] Velde C. (1999). An alternative conception of competence: implications for vocational education. J. Vocat. Educ. Train. 51, 437–447. 10.1080/13636829900200087

[B86] Wehmeyer M. L. Palmer S. B. (2003). Adult outcomes for students with cognitive disabilities three-years after high school: the impact of self-determination. Education and training in developmental disabilities. 38(2),131–144. JSTOR, www.jstor.org/stable/23879591. Accessed 29 Mar. 2021.

[B87] Westhead P. Wright M. McElwee G. (2011). Entrepreneurship: Perspectives and Cases. London: Pearson.

[B88] Wolf A. (1994). La medición de la competencia: la experiencia del Reino Unido. Revista Europea de Formación Profesional. 1, 31–37.

[B89] Wood C. Leighton D. (2010). Measuring social value: The gap between policy and practice. London: Demos.

