# Supplementary material for: Entrepreneurship for People With Disabilities: From Skills to Social Value
Source: Front Psychol. 2021 Jul 7;12:699833. doi: 10.3389/fpsyg.2021.699833 (PMC8292768; doi:10.3389/fpsyg.2021.699833)
Supplement: Supplementary file 1 [file Data_Sheet_1.pdf]

ANNEX 1. Questionnaire

Welcome to the Disability and Entrepreneurship Questionnaire! We welcome you to the questionnaire on disability and entrepreneurship that is part of the Project: DISABILITY AND ENTREPRENEURSHIP. COMPETENCY ANALYSIS (CSO2016-75818-R), of the State R&D&I Program of the Ministry of Economy and Competitiveness. The aim is to know the skills that promote and / or, on the contrary, limit the entrepreneurial activity of people with disabilities.

The questionnaire contains several blocks referring to different topics of interest for research. Please take 10 minutes of your time to complete the questionnaire.

We thank you in advance for your collaboration as your answers will be of great help to the research. The anonymity and confidentiality of the answers provided is guaranteed.

This questionnaire is designed to be completed by people with physical, organic and sensory disabilities

1. DISABILITY QUESTION BLOCK

1. Indicate what type of disability you have. [Multiple answer]:

- ☐ Osteoarticular system
- ☐ Neuromuscular system
- ☐ Cardiovascular, immune and respiratory system
- ☐ Digestive, metabolic and endocrine system
- ☐ Genitourinary system
- ☐ Hematopoietic system
- ☐ Auditive system
- ☐ Deafblindness
- ☐ Other [Specify] ____________________________
- ☐ Don’t Know/Don’t answer

2. What degree of disability have you recognized?

- ☐ From 33% to 49%
- ☐ From 50% to 64%
- ☐ From 65% to 74%
- ☐ 75% and more
- ☐ I am in process of evaluation
- ☐ Don’t Know/Don’t answer

3. Do you perceive any benefit derived from your disability?

- ☐ Yes
- ☐ No ☐ **Go to Q4**
- ☐ Don’t Know/Don’t answer ☐ **Go to Q4**

3a. How much? (in euros)

☐

4. Do you belong to an association of people with disabilities??

- ☐ Yes
- ☐ No
- ☐ Don’t Know/Don’t answer

2. ENTREPRENEURSHIP QUESTION BLOCK

5. Do you have or are you trying to start a new business, either alone or with other people? (including some form of self-employment)

☐	Yes, I have a business	**Go to Q7**
☐	No, but I intend to have it	**Go to Q7**
☐	I had it, but it was abandoned	**Go to Q11**
☐	I don't have it, nor do I intend to because I'm not interested	**Go to Q6**
☐	I don't have it because it is impossible for me because of my disability	**Go to Q1**
☐	Don't know / Don't answer	**Go to Q21**

6. From the following aspects, indicate on a scale of 1 (Nothing) to 5 (Much) the degree of importance for NOT entrepreneuring.

	1	2	3	4	5	DK/ DA
1. Lack of confidence in myself and my abilities						
2. Training deficiencies						
3. Lack of business experience						
4. Lack of financial resources						
5. Doubts about the profitability of the business						
6. Lack of stimuli in my immediate environment (family, friends, associations)						
7. Lack of institutional support (state and regional Administration)						

Go to Q14

7. What activity is / would be dedicated mainly to the company that you? Have you created / intend to create (CNAE)? [Show APPENDIX]

[ ]

8. Is / will the ownership and management of this business?

- ☐ Only mine
- ☐ Mine and my family
- ☐ Mine and other people with disabilities
- ☐ Other cases [Specify] ______________________________
- ☐ Don't know / Don't answer

9. How old is your company?

- ☐ It has not yet been launched
- ☐ Up to 3 months
- ☐ From 3 to 42 months
- ☐ More than 42 months
- ☐ Don't know / Don't answer

10. At these moments, how many people, not counting the owners, are working in this business?

[ ]

11. What is the main reason you started / plan to start?

- ☐ By necessity
- ☐ By opportunity
- ☐ Don't know / Don't answer

12. Which of the following reasons, do you think, is the MOST IMPORTANT for entrepreneurial purposes?

- [ ] Achieve greater personal independence
- [ ] Increase your personal income
- [ ] Achieve social recognition
- [ ] Achieve a personal challenge
- [ ] Other [Specify] ________________________________
- [ ] Don't know / Don't answer

13. What do you consider to be the main reason for abandoning the activity?

- [ ] Opportunity to sell the business
- [ ] Lack of profitability
- [ ] Problems obtaining financing
- [ ] Another job or business opportunity
- [ ] Retirement
- [ ] Personal reasons
- [ ] An incident (fire, natural disaster, etc.)
- [ ] Another case [Specify]] ______________________________
- [ ] Don't know / Don't answer

3. COMPETENCIES QUESTION BLOCK

14. From he following statements, related to entrepreneurship, on a scale of 1 to 5, taking into account that 1 is "Nothing" and 5 is "Much", indicate your degree of agreement:

	1	2	3	4	5	DK/ DA
I am aware of my own emotions and the effects they can have						
I know my own strengths and personal limitations						
I am a self-confident person						
I control my emotions and negative impulses						
I take my own values into account when acting						
I adapt myself to the changes						
I set high / demanding goals						
I have initiative in the face of the opportunities that present themselves						
I consider myself an optimistic personponerse en la piel del otro						
I have the ability to put myself in the "shoes of the another"						
I have the ability to interpret the emotions of others						
In my work I anticipate and recognize the needs that satisfy my clients						
I am able to detect the personal development needs of others						
I boost the personal growth of others						
I drive change in my organization / company						
I am persuasive with the activities I propose						
I manage conflicts by looking for a negotiated way to reach an agreement						
I work in team						

4. IDENTIFICATION QUESTION BLOCK

15. Place of residence:

[________________________________]

16. Sex:

- [ ] Male
- [ ] Female

17. Age:

[ ]

18. What are the highest official level studies that you? has attended (regardless of whether they have been completed or not):

- [ ] No studies
- [ ] Primary
- [ ] Compulsory secondary
- [ ] Vocational training Intermediate Level
- [ ] Baccalaureate
- [ ] Vocational training Higher Level
- [ ] University studies
- [ ] Don't know / Don't answer

19. Home situation:

- [ ] I live alone
- [ ] I live with my parents and / or other relatives
- [ ] I live with my partner
- [ ] I live with my partner and children
- [ ] I live alone with my children
- [ ] Don't know / Don't answer

20. What is the main work activity?

- [ ] Employer (employer with employees)
- [ ] Self-employed worker or employer without employees
- [ ] Public sector employee
- [ ] Private sector employee
- [ ] Unemployed
- [ ] Inactive (student, unpaid housework…)
- [ ] Don't know / Don't answer

21. Do you believe that within a year the possibility of undertaking for people with disabilities will be better, equal or worse than now?

- [ ] Better
- [ ] Same
- [ ] Worse
- [ ] Don't know / Don't answer

22. Do you think that telecommuting (or working from home) would be more suitable for the development of a business project?

- [ ] Yes
- [ ] No
- [ ] Don't know / Don't answer

23. If you want to get the overall results of the study, then check the email account you want to be sent to:

[ ]
